# Progression to kidney failure in ADPKD: the PROPKD score underestimates the risk assessed by the Mayo imaging classification

**DOI:** 10.3389/fmed.2024.1470309

**Published:** 2024-11-07

**Authors:** Daniela Maria Allmer, Diego Parada Rodriguez, Christof Aigner, Franco Laccone, Mato Nagel, Sylvia Metz-Schimmerl, Gere Sunder-Plassmann

**Affiliations:** ^1^Division of Nephrology and Dialysis, Department of Medicine III, Medical University of Vienna, Vienna, Austria; ^2^Center for Pathobiochemistry and Genetics, Institute of Medical Genetics, Medical University of Vienna, Vienna, Austria; ^3^Center for Nephrology and Metabolic Medicine, Weißwasser, Germany; ^4^Department of Biomedical Imaging and Image-Guided Therapy, Division of General and Paediatric Radiology, Medical University of Vienna, Vienna, Austria

**Keywords:** autosomal dominant polycystic kidney disease, *PKD1*, *PKD2*, disease progression, imaging classification of ADPKD, Mayo imaging classification, PROPKD score, risk assessment

## Abstract

Autosomal-dominant polycystic kidney disease (ADPKD) is the most common inherited kidney disease and fourth leading cause for renal replacement therapy worldwide. Disease progression is tightly linked to genotype, however, factors like genetic modifiers and environmental factors are responsible for a high phenotypic variability within- as well as between families. Individual’s risk of progression to kidney failure is assessed using prediction- or risk-assessment tools such as the predicting renal outcomes in ADPKD score (PROPKD score) and the Mayo Imaging Classification (MIC). The PROPKD score encompasses genetic and phenotypic parameters, while the MIC relies on renal imaging, height, and age of patients. Both methods categorize patients into low-risk, intermediate-risk, and high-risk for progression to kidney failure. In this retrospective, cross-sectional study, we calculated the risk of progression to kidney failure in our population and analyzed the agreement between the methods in three separate models with alternating stratification of MIC risk categories. We found a mismatch for risk assessment between the respective risk categories, indicating that the PROPKD score and MIC should not be used interchangeably. Preferably, the MIC should be used as a base for risk assessment and may be enhanced by genotypic and phenotypic information.

## 1 Introduction

Autosomal-dominant polycystic kidney disease (ADPKD) is the most common inherited kidney disease and fourth leading cause for renal replacement therapy worldwide (1, 2). The incidence amounts to approximately 1 in 400 to 1 in 1000. ADPKD typically presents with bilateral formation and expansion of multiple, fluid-filled cysts, progressively deforming and displacing healthy kidney tissue, eventually leading to kidney failure (3).

ADPKD is a monogenic disease inherited in an autosomal-dominant fashion, typically caused by mutations in either *PKD1* (80–85%) or *PKD2* (15–20%) (4–8). Disease caused by mutations in *PKD1* is usually correlated with a worse outcome and kidney failure at a mean age of 56 years (55.6 years if the genetic variant is truncating, versus 67.9 years if non-truncating), as opposed to the *PKD2* phenotype with a mean age of 77 years at onset of kidney failure (9, 10).

Truncating mutations are associated with more severe disease, as they are likely fully inactivating. Non-truncating variants, comprising missense variants and in-frame indels, are mostly correlated with less severe phenotypes. Inactivating homozygous mutations in *PKD1* and *PKD2* are embryonically lethal, however, *PKD1* and *PKD2* homozygous and *PKD1* compound heterozygous individuals have been identified (11–13). When both alleles are hypomorphic, the phenotype can range from typical to severe, whereas in combination with an inactivating allele, patients are more likely to develop early-onset disease. Heterozygous hypomorphic patients typically present with a weak phenotype (13).

Predicting the course of the disease and identification of rapidly progressive patients is of clinical interest, as it aids in selecting patients for specific therapies and for enrollment in clinical trials. Furthermore, many patients themselves request information regarding their risk for kidney failure. For such purposes, prediction tools or risk assessment tools are employed. The Predicting Renal Outcomes in ADPKD score, PROPKD score, is based on genetics, sex and the presence of hypertension and urologic events, while the Mayo Imaging Classification, MIC, is based on renal imaging and measurement of patient’s kidney volume by height and age (14, 15).

In practice, both the PROPKD score and the MIC are widely used prediction tools. However, neither of these methods is used consistently and the concordance of both methods is not well described. Therefore, with regard to the estimated risk of decline of kidney function, we analyzed the agreement of risk assessment using the PROPKD score and the MIC to uncover a potential redundancy or discrepancy using three different MIC risk category models.

## 2 Materials and methods

### 2.1 Study design

This is a retrospective cross-sectional study of patients with an established diagnosis of ADPKD. We describe genetic variants in *PKD1* and *PKD2*, the PROPKD score, the total kidney volume (TKV), the height-adjusted total kidney volume (htTKV) as well as the MIC and the creatinine-based estimated glomerular filtration rate (eGFR_cr_) at the time of kidney imaging.

### 2.2 Setting and participants

We used the institutional ADPKD registry of the Division of Nephrology and Dialysis, Department of Medicine III, at the Medical University of Vienna, to identify adult patients of both sexes with an established genetic diagnosis of ADPKD and CT or MRI scans of both kidneys. As such, inclusion criteria comprised a genetic test result confirming the diagnosis of ADPKD, availability of CT or MRI scans, and estimates of kidney function at the time of imaging. We excluded patients aged less than 18 years. Demographic and disease specific data from the years 2011 to 2021 were obtained from paper-based or electronic health records of our institution.

The institutional review board (IRB) at the Medical University of Vienna approved the study (unique IRB identifier: 1919/2019).

### 2.3 Kidney function

We describe kidney function with patient’s eGFR_cr_, calculated using the CKD-EPI 2021 equation (16). CKD stages (G1, G2, G3a, G3b, G4, G5) were defined according to the most recent Kidney Disease Improving Global Outcomes (KDIGO) guideline (26).

### 2.4 PROPKD score

The PROPKD score predicts renal outcomes in patients with ADPKD (14). It is a scoring system, from 0 to 9 points, as follows: being male: 1 point; a diagnosis of hypertension before 35 years of age: 2 points; first urologic event (hematuria, cyst infection or ADPKD-associated flank pain) before 35 years of age: 2 points; *PKD2* mutation: 0 points; non-truncating *PKD1* mutation: 2 points; truncating *PKD1* mutation: 4 points. Genetic testing was conducted either by sequencing all coding exons and flanking introns of the genes *PKD1* and *PKD2*, or by next-generation sequencing of a disease-specific exome panel. MLPA-analysis was utilized to detect potential large rearrangements. The three risk categories are defined as low-risk (0–3 points), intermediate-risk (4–6 points), and high-risk (7–9 points) of progression to end-stage kidney disease (ESKD) at 60 years, with corresponding risk percentages of 19.3, 60.8 and 91.9%, respectively.

### 2.5 Mayo imaging classification

The MIC was developed to predict the rate of decline of eGFR according to the htTKV at a given age (15). Total kidney volume and htTKV are calculated using a tool provided by the Mayo Clinic College of Medicine.^1^ Patients are assigned one of five classes ranging from 1A to 1E. In the original stratification, 1A corresponds to low-risk, 1B to intermediate-risk, and 1C, 1D and 1E are associated with high risk of progression to kidney failure (15). In a subsequent publication, the authors refined this stratification after discovering an association with TKV increase and eGFR decline in a smaller population of patients in classes 1D and 1E. Therefore, class 1C was merged with class 1B to form the intermediate risk category. The fact that class 1A corresponds to low-risk, and 1D and 1E to high risk of progression to kidney failure remained unchanged (17).

### 2.6 Statistical methods

Categorical variables were described as counts and frequencies and continuous variables as median and the 25th and 75th percentile. The Kruskal-Wallis-Test was used to calculate the difference between the eGFR_cr_ for *PKD1* truncating*, PKD1* non-truncating and *PKD2* variants as well as the PROPKD score of *PKD1* truncating*, PKD1* non-truncating and *PKD2* variants. The Mann–Whitney-test was used to further analyze the difference between each group. This same test was employed to compare TKV and htTKV values between men and women as well as *PKD1* and *PKD2*. We used the Kruskal-Wallis-Test for comparison of the eGFR_cr_ between the five MIC categories and to test for differences in TKV and htTKV in *PKD1* and *PKD2* when dividing *PKD1* by mutation type.

To compare risk assessment that encompasses genetic parameters (PROPKD score) with risk assessment that relies on renal imaging (MIC), we analyzed the agreement of low-risk, intermediate-risk, and high-risk categories of both prediction tools using three different agreement models. In these models, we kept the PROPKD risk categories unchanged (low-risk, 0–3 points; intermediate-risk, 4–6 points; and high-risk, 7–9 points), but grouped the MIC categories differently: In the first model low-risk corresponds to MIC 1A, intermediate-risk to MIC 1B and 1C, and high-risk to MIC 1D and 1E (17). In the second model, we grouped MIC 1A and 1B to form the low-risk MIC category, while intermediate-risk solely comprises MIC 1C, and high-risk, MIC 1D and 1E. The third model used MIC 1A to define low risk, MIC 1B to define intermediate risk, whereas MIC 1C, 1D, and 1E comprised the high-risk group (15).

The mean ages between the agreement groups were analyzed using ordinary one-way ANOVA after confirmation of normality for models 1 and 2; for model 3 a Kruskal-Wallis test was employed.

For the comparison of the proportions of similarly categorized patients using PROPKD and MIC between the three models we employed the Fisher’s exact test.

Statistical analyses were performed using GraphPad Prism Version 10.2.1.

## 3 Results

### 3.1 Patients

We enrolled 69 patients with genetic variants in *PKD1* or *PKD2*, and CT or MRI scans of both kidneys, as well as estimates of kidney function at the time of imaging, in this study. Among 185 patients with genetically confirmed ADPKD, we excluded 116 because of absent or insufficient imaging data or lack of GFR estimates at the time of imaging. Important demographic and clinical characteristics are summarized in Table 1.

**Table 1:** Demographic and clinical characteristics of 69 patients with ADPKD.

Characteristic	All	*PKD1*	*PKD2*
Count	69	54	15
Age, years	44.7 ± 9.4	44.4 ± 9.1	46.7 ± 11.1
Sex, female	38 (55.1)	31 (57.4)	7 (46.7)
Serum creatinine, mg/dl	1.2 (1.0, 1.7)	1.2 (0.9, 1.6)	1.1 (1.0, 1.7)
eGFR_cr_, ml/min per 1.73 m^2^	67.0 (45.0, 93.0)	65.5 (47.3, 89.8)	80.0 (39.5, 93.0)
CKD G1 (≥90)	20 (29.0)	14 (25.9)	6 (40.0)
CKD G2 (60–89)	18 (26.1)	16 (29.6)	2 (13.3)
CKD G3a (45–59)	14 (20.3)	12 (22.2)	2 (13.3)
CKD G3b (30–44)	11 (15.9)	7 (13.0)	4 (26.7)
CKD G4 (15–29)	5 (7.2)	4 (7.4)	1 (6.7)
CKD G5 (<15)	1 (1.4)	1 (1.9)	0 (0.0)
PROPKD score	5 (3, 6)	5 (4, 6)	2 (1, 3)
TKV, ml	1,510 (772, 2,222)	1,565 (856, 2,280)	945 (656, 1,935)
htTKV, ml/m	803 (452, 1,268)	909 (491, 1,286)	537 (357, 1,071)
Mayo imaging classification			
1A	6 (8.7)	3 (55.6)	3 (20.0)
1B	21 (30.4)	15 (27.8)	6 (40.0)
1C	17 (24.6)	13 (24.1)	4 (26.7)
1D	20 (29.0)	18 (33.3)	2 (13.3)
1E	5 (7.2)	5 (9.3)	0 (0.0)

Data are given as mean ± SD, count (%) or as median (p25, p75).

*Includes a patient with one likely benign splicing and one pathogenic missense variant in *PKD1*.

ADPKD, autosomal-dominant polycystic kidney disease; eGFR_cr_, estimated glomerular filtration rate using creatinine; CKD, chronic kidney disease; PROPKD, predicting renal outcomes in ADPKD score; TKV, total kidney volume; htTKV, height-adjusted total kidney volume.

### 3.2 *PKD1* and *PKD2* variants

Among the study participants 54 out of 69 patients (78.3%) showed genetic variants in *PKD1* (35 truncating, 50.7%, and 19 non-truncating, 27.5%) and 15 (21.7%) patients had genetic variants in *PKD2*. Table 2 gives an overview of the mutation types and the distribution among female and male study participants. Individual genetic variants including the classification according to American College of Medical Genetics and Genomics (ACMG) are presented in Supplementary Table S1.

**Table 2:** Types of genetic variants in *PKD1* and *PKD2.*

Genetic variant	All patients	Female	Male
	*N* = 69	*N* = 38	*N* = 31
*PKD1* truncating	35 (50.7)	19 (27.5)	16 (23.2)
*PKD1* non-truncating	19 (27.5)	12 (17.4)	7 (10.1)*
*PKD2*	15 (21.7)	7 (10.1)	8 (11.6)

Data are given as count (%).

*Includes a patient with one likely benign splicing and one pathogenic missense variant in *PKD1*.

### 3.3 Kidney function

**Figure 1:**
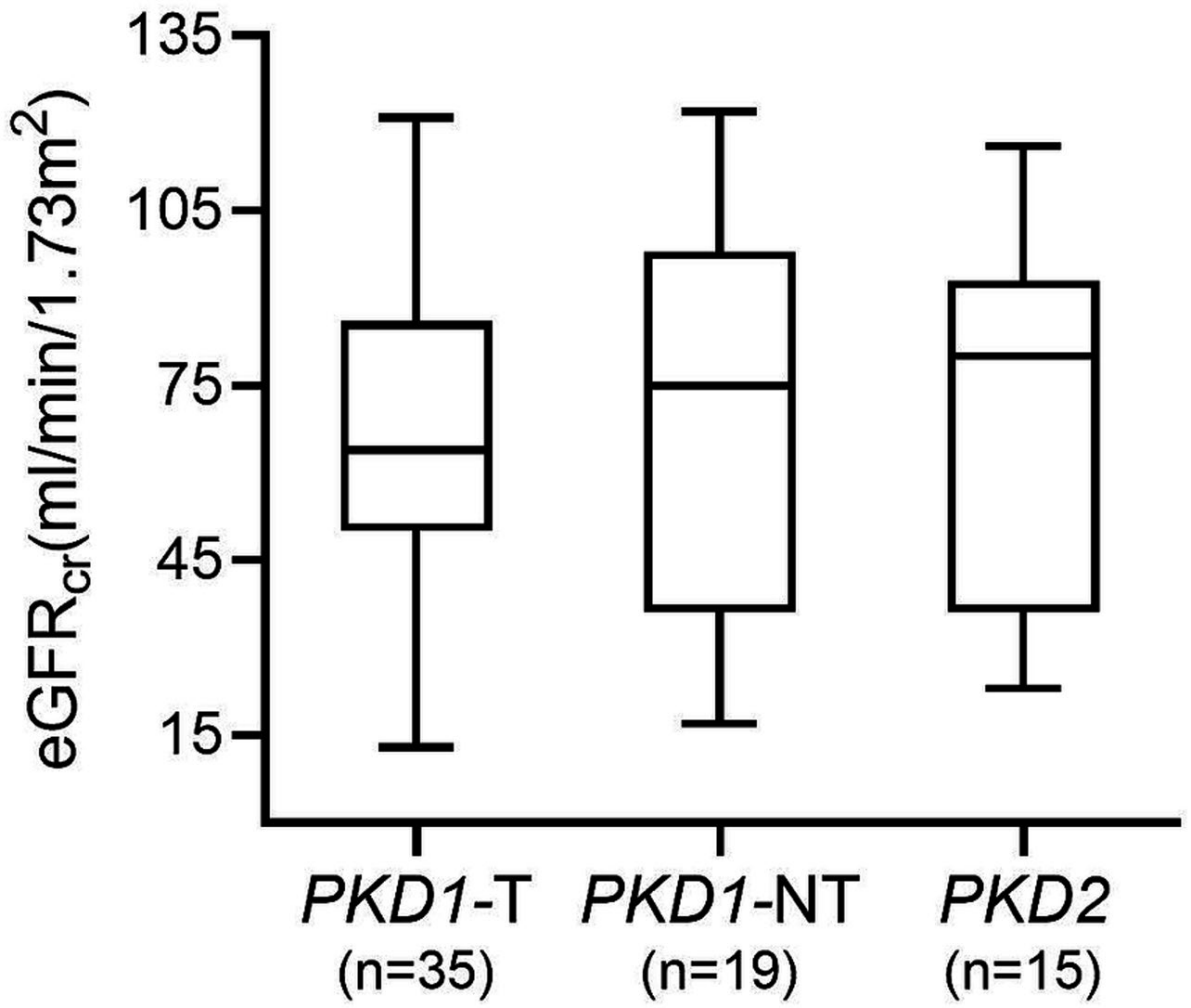
GFR estimates in patients with genetic variants in *PKD1* and *PKD2*. No significant difference between groups was found (*p* = 0.99). *PKD1*-T, *PKD1* truncating; *PKD1*-NT, *PKD1* non-truncating.

The median serum creatinine of all patients was 1.2 mg/dL (p25: 1.0, p75: 1.7) and the corresponding median eGFR_cr_ was 67.0 (p25: 45.0, p75: 93.0) ml/min per 1.73 m^2^. Figure 1 shows the eGFR of patients with genetic variants in *PKD1* (divided into truncating and non-truncating) and *PKD*2. There was no significant difference in GFR estimates between the patient groups (*p* = 0.99).

### 3.4 Total kidney volume

The median TKV and htTKV of all study participants was 1,510 mL (p25: 772, p75: 2,222) and 803 mL/m (p25: 452, p75: 1,268), respectively. In Table 3 we provide TKV and htTKV according to genotype and sex. Median TKV was 933 mL (p25: 607, p75: 2,018) in women and 1,746 mL (p25: 1,166, p75: 2,409) in men. Median htTKV was 570 mL/m (p25: 354, p75: 1,201) in women and 933 mL/m, (p25: 669, p75: 1,292) in men. Both were significantly different between men and women (TKV *p* = 0.007 and htTKV *p* = 0.015). Analyses by genotype and mutation type did not yield statistically significant results (data not shown).

**Table 3:** TKV and htTKV according to genetic variants in *PKD1* and *PKD2.*

Genetic variant	TKV	TKV	TKV	htTKV	htTKV	htTKV
	All	Female	Male	All	Female	Male
All	1,510 (772, 2,222)	933 (607, 2,018)**	1,746 (1,166, 2,409)	803 (452, 1,268)	570 (354, 1,201)***	933 (669, 1,292)
*PKD1*-T	1,761 (1,104, 2,333)	1,523 (793, 2,073)	1,965 (1,522, 2,549)	1,042 (605, 1,316)	912 (447, 1,247)	1,107 (802, 1,393)
*PKD1*-NT*	1,151 (732, 2,012)	876 (551, 1,343)	1,586 (1,249, 2,719)	669 (412, 1,121)	511 (328, 813)	906 (703, 1,464)
*PKD2*	945 (656, 1,935)	702 (496, 1,517)	1,436 (874, 1,841)	537 (357, 1,071)	450 (292, 909)	807 (494, 1,012)

Data are given as median (p25, p75).

*Includes 1 patient with one likely benign splicing and one pathogenic missense variant in *PKD1*.

**p = 0007 all females vs. all males.

***p = 0.015 all females vs. all males.

*PKD1-T*, *PKD1* truncating; *PKD1-NT*, *PKD1* non-truncating; TKV, total kidney volume (ml); htTKV, height-adjusted total kidney volume (ml/m).

### 3.5 PROPKD score

**Figure 2:**
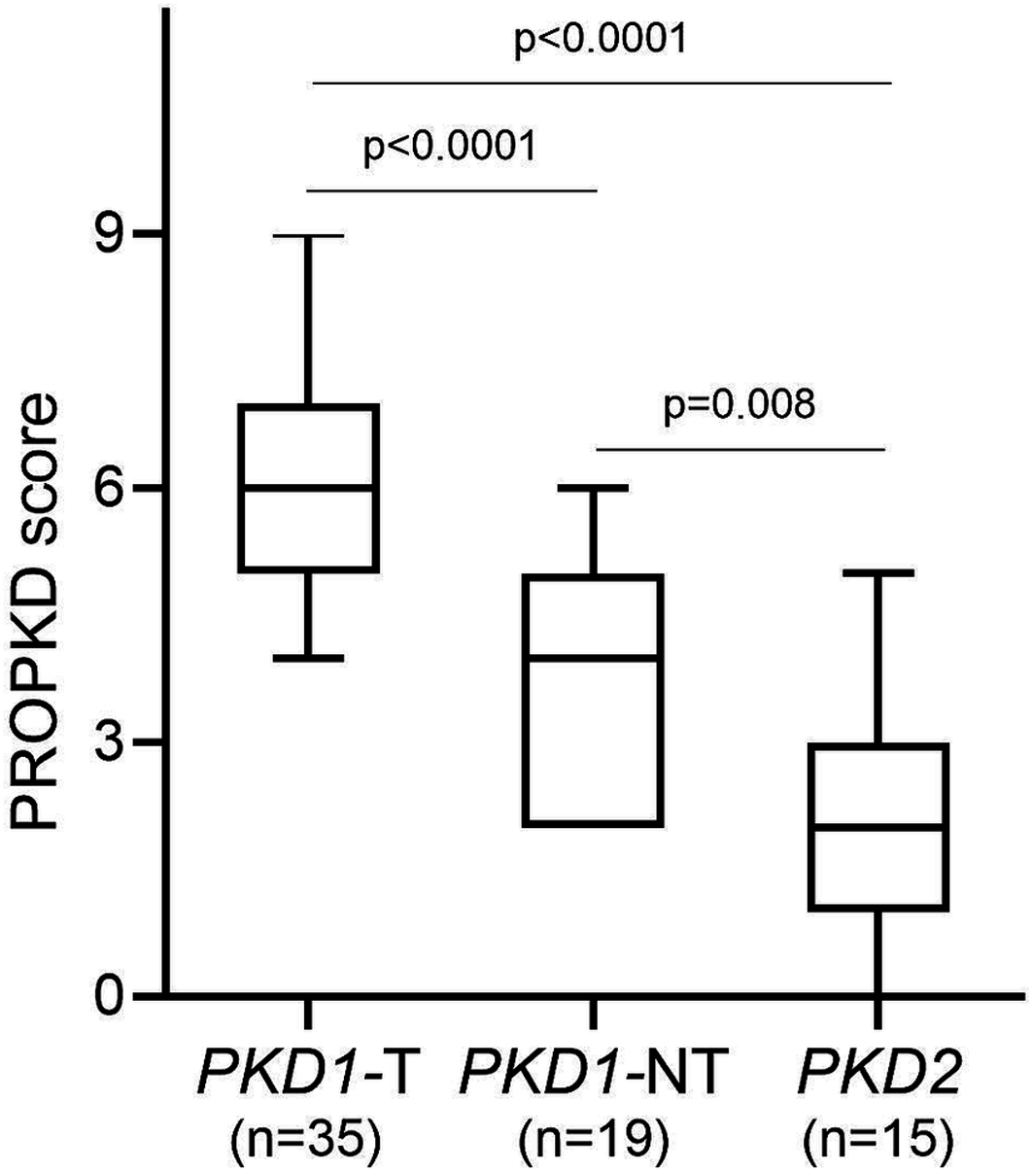
PROPKD score according to genetic variants in *PKD1* and *PKD2*. *PKD1*-T, *PKD1* truncating; *PKD1*-NT, *PKD1* non-truncating.

**Figure 3:**
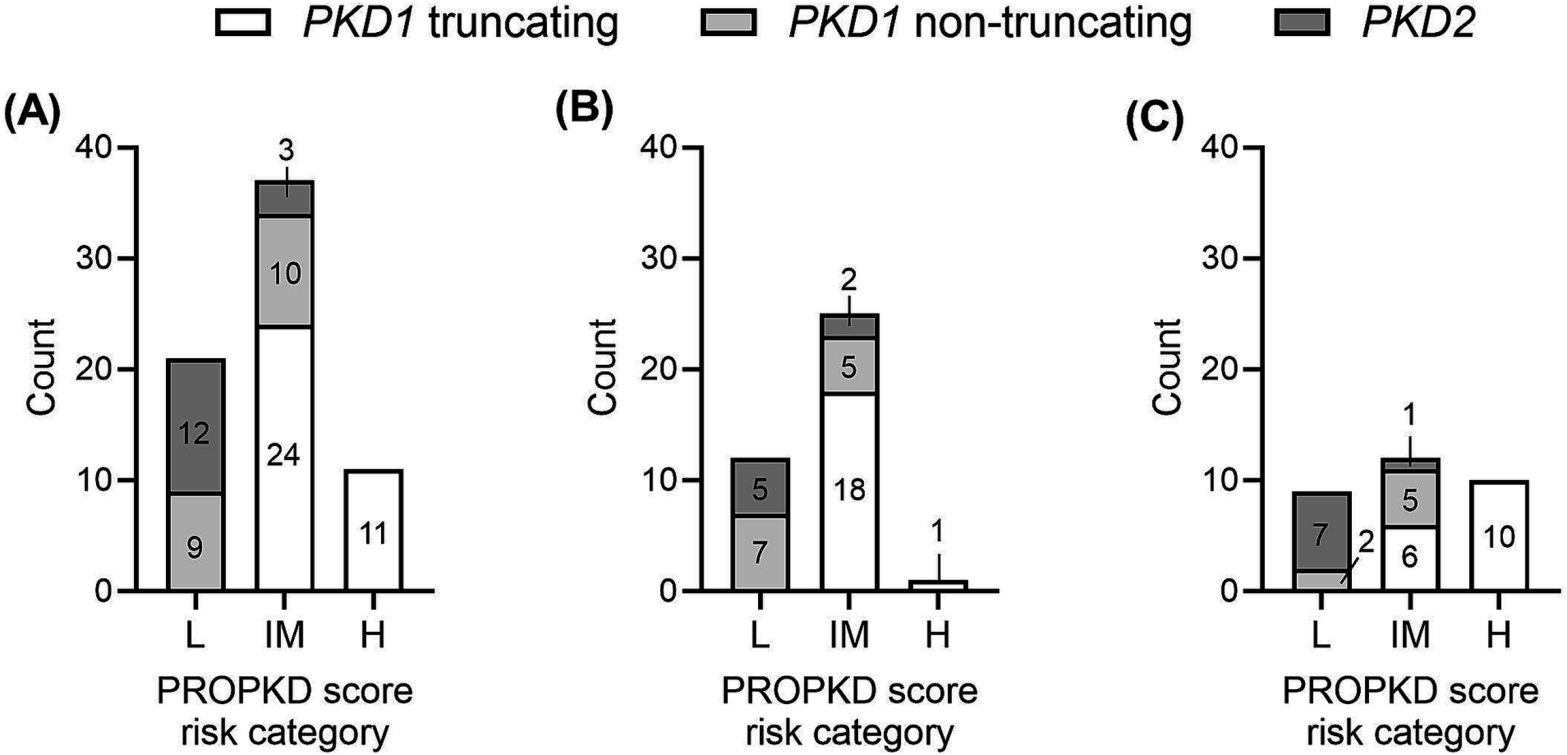
Distribution of types of genetic variants (*PKD1* truncating, *PKD1* non-truncating, *PKD2*) among the three PROPKD risk categories **(A)** in all patients, **(B)** in female patients, **(C)** in male patients. L, low risk; IM, intermediate risk; H, high risk.

The median PROPKD score of the study cohort as a whole was 5 (p25: 3, p75: 6), with a significant difference between *PKD1* truncating (6, p25: 5, p75: 7), *PKD1* non-truncating (4, p25: 2, p75: 5) and *PKD2* (2, p25: 1, p75: 3); *p* < 0.0001 when comparing *PKD1* truncating and *PKD1* non-truncating as well as *PKD1* truncating vs. *PKD2*; *p* = 0.008 when comparing *PKD1* non-truncating and *PKD2* (Figure 2). Twenty-one (30.4%) participants showed a low risk of progression to kidney failure, 37 (53.6%) an intermediate risk, and 11 (15.9%) a high risk. Figure 3 indicates the different ADPKD genotypes among the three PROPKD risk categories in all patients, as well as in females and in males.

### 3.6 Mayo imaging classification

**Figure 4:**
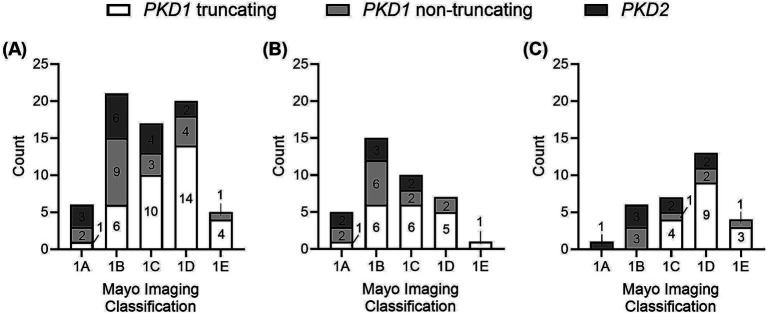
Distribution of types of genetic variants (*PKD1* truncating, *PKD1* non-truncating, *PKD2*) among the five MIC categories **(A)** in all patients, **(B)** in female patients, **(C)** in male patients.

**Figure 5:**
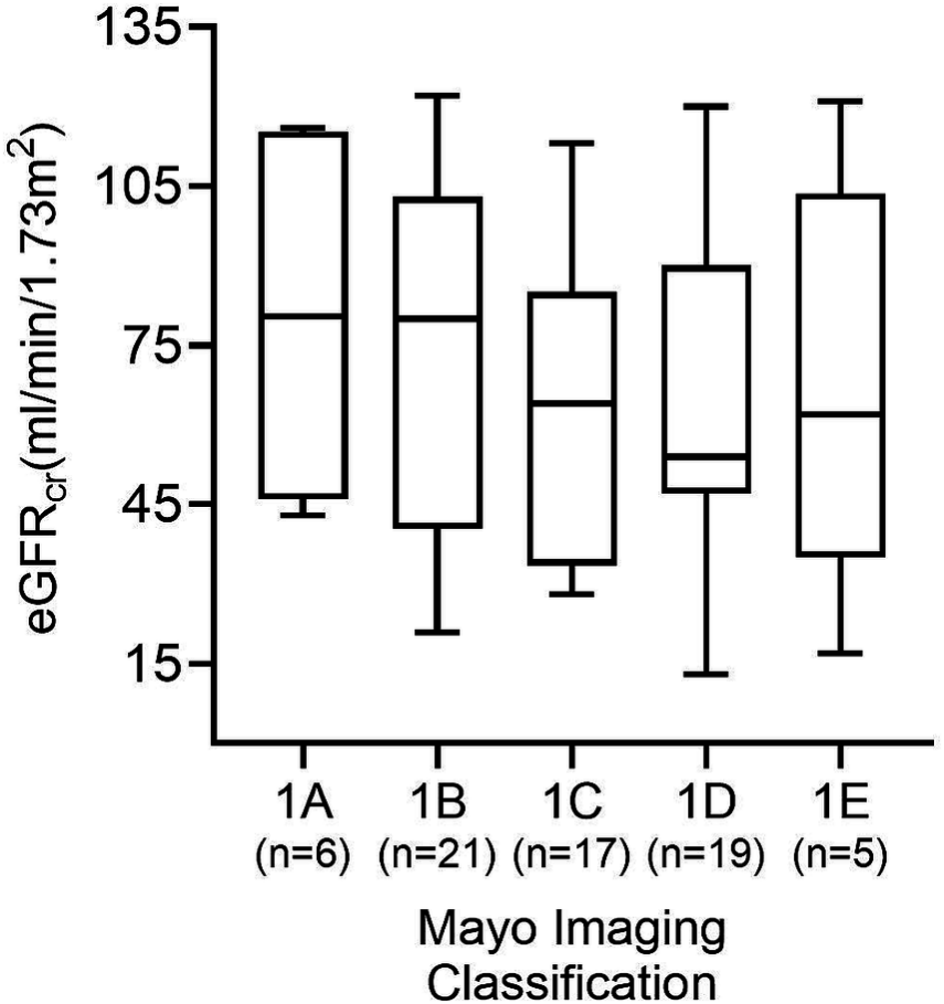
GFR estimates according to each of the five MIC categories in all 69 patients. No significant difference between groups was found (*p* = 0.48).

Six (8.7%) patients were categorized in MIC class 1A, 21 (30.4%) in MIC 1B, 17 (24.6%) in MIC 1C, 20 (29.0%) in MIC 1D, and 5 (7.2%) in MIC 1E. Figure 4 displays the ADPKD genotype distribution in the five MIC categories of all patients and of females and males. The eGFR of all 69 study participants according to MIC category is shown in Figure 5, without statistical significance between groups (*p* = 0.48).

### 3.7 Agreement of the PROPKD score and the Mayo imaging classification

**Figure 6:**
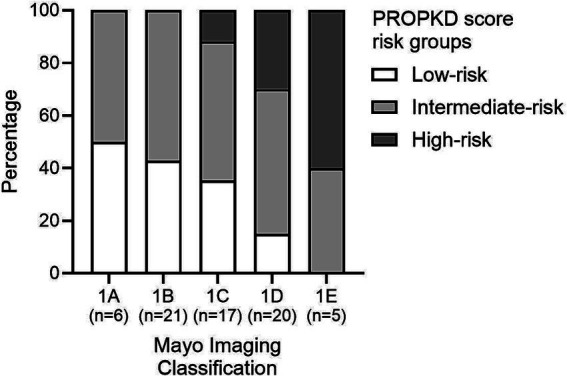
Distribution of the three PROPKD risk categories among the five Mayo imaging classes.

Figure 6 shows the distribution of PROPKD risk categories among the five different MIC groups. The proportion of PROPKD low-risk patients decreases with higher MIC, while the proportion of intermediate- and high-risk patients increases. The stratification of the MIC risk categories has been changed over time, thus analysis of the agreement of different approaches seemed reasonable.

### 3.8 Agreement model 1

Analyzing the first model, using the current, refined MIC stratification (17) we found that out of a total of 21 patients in the PROPKD low-risk group, only 3 corresponded to MIC 1A (14.3%). 9 patients fell into MIC 1B, 6 into 1C, and 3 into 1D. The total number of patients in MIC 1A was 6, so 50.0% agreed with the low-risk PROKPD the other way around. The remaining 3 patients with MIC 1A fell into the intermediate-risk group with a PROPKD score of 4.

Out of a total of 37 patients with intermediate-risk PROPKD, 21 agreed with an MIC of 1B or 1C (56.8%). The remaining 16 patients separated into low-risk MIC 1A (*n*=3), and high-risk 1D (*n*=11) and 1E (*n*=2). Conversely, 38 patients had MIC 1B or 1C and the 21 patients corresponded to an agreement of 55.3% with an intermediate-risk PROPKD score of 4–6. Out of the 17 remaining patients, 15 corresponded to low risk, whereas 2 fell into high-risk PROPKD.

**Figure 7:**
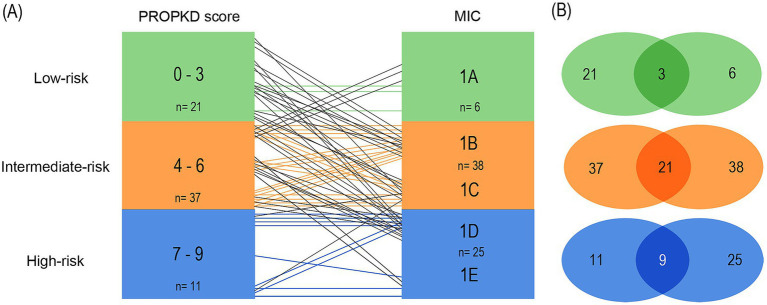
Agreement of kidney failure risk categories defined by the PROPKD score and the MIC in 69 patients with ADPKD, model 1. Low-risk in green color (PROPKD score 0 to 3, MIC 1A), intermediate-risk in orange color (PROPKD score 4 to 6, MIC 1B and 1C), and high-risk in blue color (PROPKD score 7 to 9, MIC 1D and 1E). **(A)** Agreement between risk categories is indicated by colored lines, disagreement by black lines. **(B)** Numbers in the middle indicate the count of patients showing agreement of kidney failure risk categories.

Lastly, 9 out of 11 patients (81.8%) in PROPKD high-risk agreed with MIC 1D or 1E, while the remaining two had MIC 1C. On the other hand, only 9 out of 25 patients (36.0%) with MIC 1D or 1E also had a PROPKD score of 7–9. The others fell into low-risk (*n*=3) and intermediate-risk (*n*=13) groups (Figures 7A,B).

These findings imply that the PROPKD score may tend to underestimate risk of progression when compared to MIC and actual renal volume, since 33/69 (47.8%) patients agreed with their corresponding MIC risk group, 5 were overestimated (7.2%) and 31 were underestimated (44.9%).

### 3.9 Agreement model 2

When pairing classes 1A and 1B to form the low-risk MIC category, we found 12/21 (57.1%) agreement of low-risk PROPKD with MIC 1A and 1B. The 9 remaining patients had MIC 1C (*n*=6) and 1D (*n*=3). 9/37 (24.3%) of the intermediate PROPKD score agreed with MIC 1C, while 15 patients segregated into low-risk groups 1A (*n*=3) and 1B (*n*=12) and 13 into high-risk groups 1D (*n*=11) and 1E (*n*=2). Agreement between high-risk PROPKD and MIC remained unchanged compared to model 1.

Conversely, in 12/27 (44.4%) patients we found agreement of MIC 1A and1B with low-risk PROPKD, while the remaining 15 patients presented with an intermediate-risk PROPKD score. 9/17 (52.9%) of MIC 1C agreed with intermediate-risk PROPKD, the other patients scored for low risk (*n*=7) and high risk (*n*=2). Again, the agreement between high-risk PROPKD and MIC remained unchanged.

**Figure 8:**
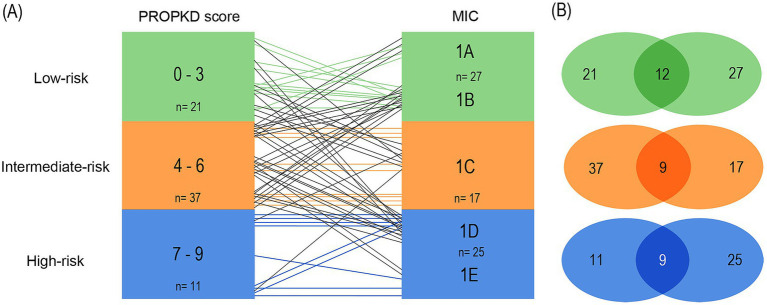
Agreement of kidney failure risk categories defined by the PROPKD score and the MIC in 69 patients with ADPKD, model 2. Low-risk in green color (PROPKD score 0 to 3, MIC 1A and 1B), intermediate-risk in orange color (PROPKD score 4 to 6, MIC 1C), and high-risk in blue color (PROPKD score 7–9, MIC 1D and 1E). **(A)** Agreement between risk categories is indicated by colored lines, disagreement by black lines. **(B)** Numbers in the middle indicate the count of patients showing agreement of kidney failure risk categories.

This leads to a total agreement of 30/69 (43.5%), with 17/69 (24.6%) patients left overestimated and 22/69 (31.9%) underestimated for this arrangement, which, besides a lower overall agreement, renders the disagreement more balanced when compared to the first model (Figures 8A,B).

### 3.10 Agreement model 3

**Figure 9:**
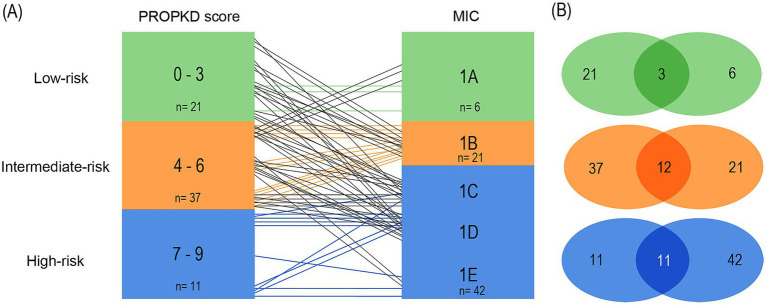
Agreement of kidney failure risk categories defined by the PROPKD score and the MIC in 69 patients with ADPKD, model 3. Low-risk in green color (PROPKD score 0–3, MIC 1A), intermediate-risk in orange color (PROPKD score 4 to 6, MIC 1B), and high-risk in blue color (PROPKD score 7 to 9, MIC 1C, 1D and 1E). **(A)** Agreement between risk categories is indicated by colored lines, disagreement by black lines. **(B)** Numbers in the middle indicate the count of patients showing agreement of kidney failure risk categories.

In the third model (Figures 9A,B), we included the stratification of the initial publication of the MIC in our analyses (15). Class 1A comprised low-risk patients, 1B intermediate-risk and 1C to 1E high-risk patients with likely rapidly progressive disease.

3/21 (14.3%) patients agreed in low-risk by both methods, while 9 patients with low-risk PROPKD fell into intermediate MIC 1B and 6 into high-risk MIC 1C. 3 patients had high-risk MIC 1D. 12/37 (32.4%) patients agreed in intermediate risk. Out of the remaining 25 patients, 22 with intermediate-risk PROPKD were classified as high-risk MIC, namely 9 in 1C, 11 in 1D, and 2 in 1E. 3 patients with an intermediate PROPKD score had low-risk MIC 1A. 11/11 (100.0%) patients with high-risk PROPKD scored for high-risk MIC.

Vice versa, we found 3/6 (50.0%) patients with low-risk MIC agreed with low-risk PROPKD, while the remaining 3 patients scored for intermediate-risk PROPKD. Out of 21 patients with MIC 1B, 12 likewise scored for intermediate-risk PROPKD (57.1%), while the remaining 9 patients scored for low-risk PROPKD. 11/42 (26.2%) patients with high-risk MIC agreed with high-risk PROPKD, while 22 had intermediate-risk PROPKD and 9 scored for low risk.

As such, this model’s overall agreement was 26/69 (37.7%) by both methods, with 40/69 (58.0%) patients being underestimated by the PROPKD score, and 3/69 (4.3%) overestimated.

Thus, regardless of MIC risk category arrangement, there was a notable difference between PROPKD risk categories and their contemplated MIC counterparts in our cohort, indicating that the corresponding PROPKD score and MIC risk categories may not be used interchangeably.

Comparing the proportion of patients agreeing and disagreeing by PROPKD and MIC in each model, we found the difference to be non-significant (*p* = 0.48). Individual data of patients are presented in Supplementary Table S1.

## 4 Discussion

In this study we tested the agreement of two tools for the assessment of the risk of kidney disease progression in ADPKD, the PROPKD score and the MIC, respectively. We examined patients with variants in *PKD1* or *PKD2*, presenting with a median eGFR_cr_ of 67.0 mL/min per 1.73 m^2^, a median htTKV of 803 mL/m, and a median PROPKD score of 5, using three different models of agreement. Overall, as compared to the MIC, the PROPKD score underestimated the risk of progression in the majority of patients.

The MIC or the PROPKD score are frequently used for assessment of patients with ADPKD (14, 15, 17–23). However, only few studies examined the agreement of the PROPKD score and the MIC category in these patients (18, 24).

Cornec-Le Gall et al. (18) analyzed the agreement of the PROPKD score and MIC in their cohort in 4 different groups: Group 1 comprised patients with agreement in the intermediate- and high-risk categories by both methods, Group 2 comprised low-risk patients that agreed by both methods, and Groups 3 and 4 comprised patients that were underestimated by either the PROPKD score (MIC high-risk patients) or the MIC (PROPKD high-risk patients), respectively. The authors found a high conformity in Group 1 (75.7%), and a very poor conformity in Group 2 (3.6%). Underestimation by PROPKD was found in 14.1% of cases of Group 3, while MIC underestimated 6.6% of patients in Group 4. Groups 3 and 4 had a significantly higher average age, while the fraction of patients in MIC 1C was higher in Group 3 compared to Group 1. PROPKD intermediate-risk patients were similarly more common in Group 4 than 1, which led the authors to the conclusion of milder disease progression in the respective patients.

Secondly, Naranjo and co-workers compared several different risk assessment strategies among 164 patients with ADPKD (24). By MRI, 118 patients were classified as rapidly progressing, including MIC 1C, 1D, and 1E. Regarding the PROPKD score, patients with a preliminary score of 3 or more, so without inclusion of points for genetic variants in *PKD1* or *PKD2*, qualified for subsequent genetic testing, enriching the population for higher MIC categories. A *PKD1* or *PKD2* variant was found in 64/164 (39.0%) of 68 genetically tested individuals. After this preselection, 27/164 (16.5%) had a PROPKD score > 6, and 25/27 (92.6%) a MIC category of 1C, 1D, or 1E. Notably, in 96/164 (58.5%) patients genetic testing was not performed. The authors propose that the PROPKD score is very specific but has low sensitivity.

In line with these two studies Lavu and colleagues suggested that combining genetic and imaging data is valuable in identification of rapidly progressive patients among the Mayo Clinic *PKD1*/*PKD2* population (25). However, all three aforementioned studies did not directly compare the three risk categories of MIC and PROPKD score.

In our study, we examined the agreement of the PROPKD score and the MIC risk categories using three different models of agreement: In the first model, the PROPKD low-risk score corresponds to MIC 1A, intermediate risk to MIC 1B and 1C, and high risk to MIC 1D and 1E (17). In the second model, we grouped MIC 1A and 1B to form the low-risk MIC category, while intermediate risk solely comprises MIC 1C, and high-risk MIC 1D and 1E. The third model used MIC 1A to define low risk, MIC 1B to define intermediate risk, whereas MIC 1C, 1D, and 1E comprised the high-risk group (15).

In all three models, the PROPKD score underestimated the risk as compared to the MIC and overall agreement was best in model 1 (47.8%) as compared to model 2 (43.5%) and model 3 (37.7%).

Considering the previously mentioned four patient groups in the study of Cornec-Le Gall et al. (18) we found that our agreement model 3 compared best with this study. Analyzing model 1, we found 43.5% patients belonging to Group 1, 4.3% to Group 2, 44.9% to Group 3 and 7.2% to Group 4. In comparison, it seemed that about 30% of patients were shifted from Group 1 to Group 3 in our cohort, leading to a higher disagreement. All patients in Group 3 were over 35 years of age (mean 48.1 ± 7.6 years), while 60% were in Group 4 (mean 39.0 ± 13.8 years) and the average age in Group 1 was 42.1 ± 9.6 years (*p* = 0.016). We did not find that MIC 1C was more represented in Group 3 (19.4%) as opposed to Group 1 (30.0%). Lastly, in model 1 we could not confirm that the ratio of PROPKD intermediate-risk patients was higher in Group 1 (70.0%) than in Group 4 (60.0%).

Thus, in our cohort, model 3 representing the original stratification was the only one that confirmed all points made by Le Gall et al., while model 2 had matching results for the proportion of PROPKD intermediate-risk patients in Group 4. Model 1 representing the refined MIC stratification only matched with their findings when it came to higher average ages in Groups 3 and 4.

In view of the limited published data and the results of our study we propose that the MIC employed as the foundation of risk assessment may be further enhanced by consideration of the PROPKD score. Furthermore, we recommend re-evaluation and referral of patients to kidney imaging for calculating kidney volume and MIC if previous risk assessment, follow-up intervals and possible therapy were based on evaluation by the PROPKD score.

From the patient’s point of view, mis-categorization into a better risk category may result in a false sense of safety regarding their health status, while eGFR decline and increase of TKV happen more rapidly than anticipated. Mis-categorization into a worse risk category, albeit more seldom as of our findings, may induce anxiety and negatively affect patient’s mental health. We recommend re-evaluating patients in the PROPKD low- and intermediate-risk categories that did not already undergo kidney imaging, as we found that these categories were the ones largely underestimated. Re-evaluation of patient’s MIC could be feasible in cases where patients are located in the margin zone between MIC 1B and 1C, to examine a potential treatment option with Tolvaptan. Therefore, we suggest that assessment of the eGFR and MIC is executed on a regular basis, not only to consolidate previous findings, but to furthermore investigate a potential indication for treatment with Tolvaptan, which is suitable for patients in MIC classes 1E and 1D, and may be considered for patients in class 1C with evaluation of additional factors.

A potential limitation to our study comprises the sample size, which is, however, counterbalanced by this first direct comparison of PROPKD score and MIC using three different agreement models.

In conclusion, our analysis of the agreement of MIC and PROPKD risk categories comparing three different models of agreement suggests to group MIC 1B not in the low-risk group, and MIC 1C not in the high-risk group. As such, MIC intermediate-risk (1B and 1C) corresponds numerically best to the PROPKD intermediate-risk group. However, MIC intermediate- and high-risk patients are still frequently underestimated by the PROPKD score, which should be kept in mind in the case of unavailability of kidney imaging.

## Data Availability

The raw data supporting the conclusions of this article will be made available by the authors, upon reasonable request. Requests to access these datasets should be directed to daniela.allmer@meduniwien.ac.at or gere.sunder-plassmann@meduniwien.ac.at.

## Footnotes

^1^ https://www.mayo.edu/research/documents/pkd-center-adpkd-classification/doc-20094754
