# Supplementary material for: Progression to kidney failure in ADPKD: the PROPKD score underestimates the risk assessed by the Mayo imaging classification
Source: Front Med (Lausanne). 2024 Nov 7;11:1470309. doi: 10.3389/fmed.2024.1470309 (PMC11578822; doi:10.3389/fmed.2024.1470309)
Supplement: Supplementary file 1 [file Table_1.DOCX]

**Supplemental Table 1.** Genetic variants in *PKD1* and *PKD2*, PROPKD score, and MIC of individual patients.

Family No. Patient No. Sex Gene c. p. Class ACMG PROPKD MIC

156 164 f *PKD1* 160_166dupCGCGGGC Leu56Profs*60 Frameshift P 7 1D

99 31 m *PKD1* 165_171delGCTGCGG Leu56Argfs*15 Frameshift P 6 1D

3 97 f *PKD1* 231C>G Asn77Lys Missense VUS 2 1D

3 98 m *PKD1* 231C>G Asn77Lys Missense VUS 5 1D

42 99 f *PKD1* 412C>T Arg138* Nonsense P 4 1B

146 19 m *PKD1* 800_801delAC His267Argfs*103 Frameshift P 9 1D

10 32 f *PKD1* 856_862delTCTGGCC Gly287Terfs*1 Frameshift P 6 1B

12 111 f *PKD1* 974A>G Tyr325Cys Missense LP 4 1A

12 135 f *PKD1* 974A>G Tyr325Cys Missense LP 6 1B

118 88 m *PKD1* 1117C>G Leu373Val Missense VUS 3 1B

22 41 m *PKD1* 1145G>T Gly382Val Missense VUS 5 1B

22 83 f *PKD1* 1145G>T Gly382Val Missense VUS 4 1D

44 165 f *PKD1* 1201+1G>T Splicing P 4 1B

33 105 f *PKD1* 1272_1273dupGG Glu425Glyfs*41 Frameshift P 4 1C

25 15 m *PKD1* 1401G>A Trp467* Nonsense P 5 1C

25 116 m *PKD1* 1401G>A Trp467* Nonsense P 9 1E

26 106 m *PKD1* 1547G>A Trp516* Nonsense P 9 1C

138 4 m *PKD1* 2097+1G>A Splicing P 5 1D

2 122 f *PKD1* 2129T>C Leu710Pro Missense VUS 4 1B

129 120 m *PKD1* 2661_2679del19 Trp887Cysfs*5 Frameshift P 7 1D

103 24 m *PKD1* 3922C>T Gln1308* Nonsense P 5 1D

130 178 f *PKD1* 4500G>C Trp1500Cys Missense VUS 2 1A

41 48 m *PKD1* 6090delC Val2031Trpfs*85 Frameshift P 5 1E

92 45 f *PKD1* 6533G>A Cys2178Tyr Missense LP 2 1C

30 9 f *PKD1* 6534C>G Cys2178Trp Missense VUS 2 1B

161 139 m *PKD1* 6559T>C Trp2187Arg Missense LP 5 1E

71 112 f *PKD1* 6656C>T Pro2219Leu Missense LP 4 1B

89 14 f *PKD1* 6994_7000dupGCTGGCG Val2334Glyfs*88 Frameshift P 6 1B

157 173 f *PKD1* 7288C>T Arg2430* Nonsense P 4 1A

7 39 m *PKD1* 7863+1G>T Splicing P 9 1D

145 144 m *PKD1* 7915C>T Arg2639* Nonsense P 5 1C

76 21 f *PKD1* 7973_7974delTG Val2658Glyfs*2 Frameshift P 8 1E

64 51 m *PKD1* 8017-31_8161+225del401 Large Deletion P 7 1C

100 148 f *PKD1* 8378T>G Leu2793Arg Missense VUS 2 1C

120 3 f *PKD1* 8941delT Ser2981Profs*13 Frameshift P 6 1B

116 136 f *PKD1* 9474_9475delAG Arg3158Serfs*20 Frameshift P 6 1C

54 37 m *PKD1* 9583T>C Trp3195Arg Missense VUS 5 1D

127 25 f *PKD1* 9712+1G>A Splicing P 4 1D

21 23 m *PKD1* 10151C>G Ser3384* Nonsense P 7 1D

21 55 f *PKD1* 10151C>G Ser3384* Nonsense P 6 1C

81 108 f *PKD1* 10405G>A Asp3469Asn Missense LP 2 1B

155 103 m *PKD1* 10405G>T Asp3469Tyr Missense LP 3 1C

28 66 f *PKD1* 10659G>A Trp3553* Nonsense P 4 1C

154 38 f *PKD1* 10669delG Ala3557Profs*27 Frameshift P 6 1D

72 177 m *PKD1* 10873delG Asp3625Metfs*7 Frameshift P 5 1D

125 96 m *PKD1* 11014-3C>T Splicing P 5 1B

*PKD1* 11180T>A Met3727Lys Missense VUS

158 74 m *PKD1* 11340_11344delTTACG Asp3780Glufs*34 Frameshift P 7 1D

23 78 f *PKD1* 12010C>T Gln4004* Nonsense P 6 1C

23 79 m *PKD1* 12010C>T Gln4004* Nonsense P 9 1E

147 137 f *PKD1* 12013C>T Gln4005* Nonsense P 6 1C

36 87 f *PKD1* 12404G>C Arg4135Pro Missense LP 2 1B

140 170 f *PKD1* 12489_12490delTC Arg4164Leufs*45 Frameshift P 6 1B

162 26 f *PKD1* Del Ex 22-43 Large Deletion P 6 1D

13 85 f *PKD1* Del Ex 31-33 Large Deletion P 4 1D

16 94 m *PKD2* 478C>T Gln160* Nonsense P 1 1D

85 115 m *PKD2* 592C>T Arg198* Nonsense P 1 1B

73 80 m *PKD2* 916C>T Arg306* Nonsense P 3 1C

80 36 m *PKD2* 916C>T Arg306* Nonsense P 3 1A

18 152 m *PKD2* 916C>T Arg306* Nonsense P 1 1B

18 153 m *PKD2* 916C>T Arg306* Nonsense P 5 1B

107 82 m *PKD2* 958C>T Arg320* Nonsense P 1 1D

38 5 f *PKD2* 965G>A Arg322Gln Missense P 2 1A

141 30 f *PKD2* 973C>T Arg325* Nonsense P 0 1C

110 16 f *PKD2* 1003C>T Gln335* Nonsense P 2 1B

62 171 f *PKD2* 1213C>T Gln405* Nonsense P 0 1B

93 110 f *PKD2* 1330G>C Glu444Gln Missense LP 0 1B

77 119 f *PKD2* 1392A>G crypt. Splicing VUS 4 1C

45 58 f *PKD2* 1716+1G>A Splicing P 4 1A

61 49 m *PKD2* 1898+1G>C Splicing P 3 1C

All variants were found in a heterozygous state; PROPKD score, predicting renal outcomes in ADPKD score; MIC, Mayo Imaging Classification; ACMG, American College of Medical Genetics and Genomics; P, pathogenic; LP, likely pathogenic; VUS, variant of uncertain significance
